# Nine‐banded armadillo (*Dasypus novemcinctus*) activity patterns are influenced by human activity

**DOI:** 10.1002/ece3.8257

**Published:** 2021-11-03

**Authors:** Brett A. DeGregorio, Connor Gale, Ellery V. Lassiter, Andrhea Massey, Caleb P. Roberts, John T. Veon

**Affiliations:** ^1^ U.S. Geological Survey Fish and Wildlife Cooperative Research Unit University of Arkansas Fayetteville Arkansas USA; ^2^ Department of Biological Sciences University of Arkansas Fayetteville Arkansas USA

**Keywords:** anthropogenic disturbance, behavioral plasticity, domestic dogs, facultatively nocturnal, human activity

## Abstract

As the human footprint upon the landscape expands, wildlife seeking to avoid human contact are losing the option of altering their spatial distribution and instead are shifting their daily activity patterns to be active at different times than humans. In this study, we used game cameras to evaluate how human development and activity were related to the daily activity patterns of the nine‐banded armadillo (*Dasypus novemcinctus)* along an urban to rural gradient in Arkansas, USA during the winter of 2020–2021. We found that armadillos had substantial behavioral plasticity in regard to the timing of their activity patterns; >95% of armadillo activity was nocturnal at six of the study sites, whereas between 30% and 60% of activity occurred during the day at three other sites. The likelihood of diurnal armadillo activity was best explained by the distance to downtown Fayetteville (the nearest population center) and estimated ambient sound level (both indices of human activity) with armadillos being most active during the day at quiet sites far from Fayetteville. Furthermore, armadillo activity occurred later during the night period (minutes after sunset) at sites near downtown and with higher anthropogenic sound. Anecdotal evidence suggests that the observed activity shift may be in response to not only human activity but also the presence of domestic dogs. Our results provide further evidence that human activity has subtle nonlethal impacts on even common, widespread wildlife species. Because armadillos have low body temperatures and basal metabolism, being active during cold winter nights likely has measurable fitness costs. Nature reserves near human population centers may not serve as safe harbors for wildlife as we intend, and managers could benefit from considering these nonlethal responses in how they manage recreation and visitation in these natural areas.

## Graphical abstract

The nine‐banded armadillo (*Daspyus novemcinctus*) is a widespread mammal capable of being active during the day or night. Here, we present evidence that armadillos living in proximity to humans shift their activity to be nocturnal despite likely thermal fitness costs to avoid human contact.

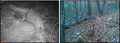

## 1 INTRODUCTION

The global expansion of human activity has had profound consequences for wildlife. While the effects of habitat destruction on species and ecosystems have been well‐documented, the indirect effects of humans on wildlife have garnered less attention (Gaynor et al., 2018). Wildlife may perceive human activity as a danger (Walther, 1969) and adopt predator avoidance strategies, primarily by choosing to avoid areas with a high risk of human contact (Frid & Dill, 2002; Tucker et al., 2018). A broad range of wildlife have been shown to physically avoid areas of high human activity such as roads and trails (Abba et al., 2007; George & Crooks, 2006; Lewis et al., 2021; Nickel et al., 2020). However, as the human footprint continues to grow, there are fewer natural areas for animals to seek refuge that are away from human activity; this is particularly true in urban or suburban areas. In these human‐dominated landscapes, wildlife must rely on other tactics to avoid contact with humans.

A growing body of evidence indicates that wildlife may shift their daily activity patterns to avoid the times of the day when human activity is highest (Kronfeld‐Schor & Dayan, 2003; Reilly et al., 2017). A recent meta‐analysis of 72 studies showed that wildlife increased their nocturnality by a factor of 1.36 times in response to human disturbance (Gaynor et al., 2018). This behavioral response may be most pronounced in mammals because many species possess the sensory capabilities and behavioral plasticity to function effectively during day or night (Kronfeld‐Schor & Dayan, 2003; Roll et al., 2006). By obtaining a better understanding of how widespread this behavioral response is within wildlife, management agencies will be able to make more informed decisions about the conservation of mammals within mixed‐use landscapes.

**FIGURE 1:**
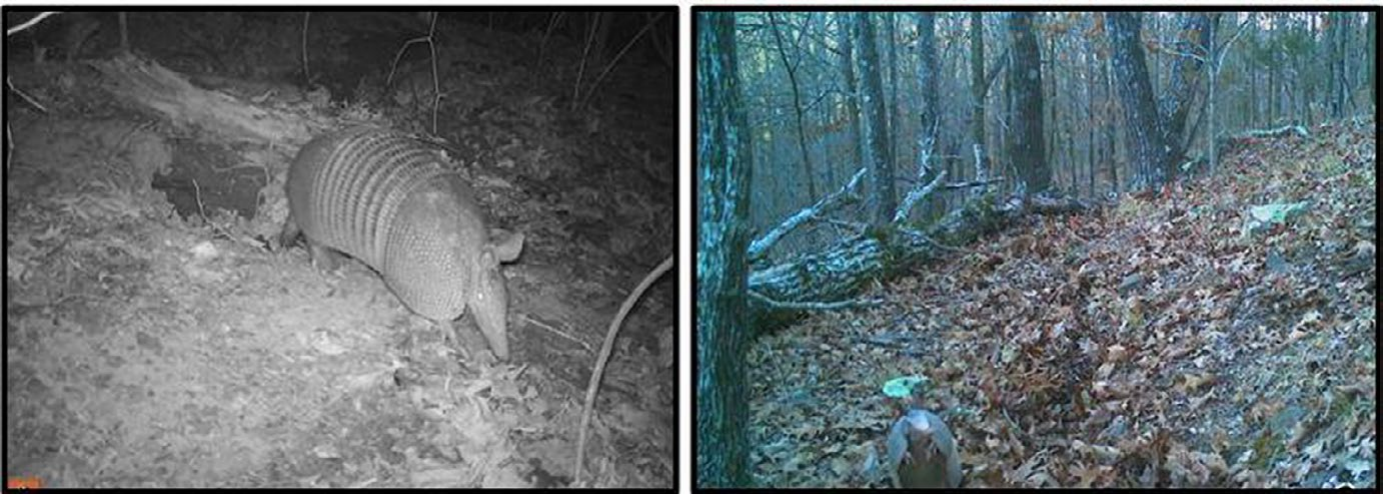
The nine‐banded armadillo (*Dasypus novemcinctus*) is a widespread mammal capable of being active during the day or night. Photographs taken by wildlife game cameras set by B.A. DeGregorio

The nine‐banded armadillo (*Dasypus novemcinctus*; hereafter, armadillo; Figure 1) is a widespread human‐tolerant species that is increasing its range across North America. During the course of unrelated field work, we noticed at some study sites that armadillos were frequently encountered during the day and at other sites armadillos were never seen even though it was known that they occurred at these locations. While the armadillo has primarily been reported to be nocturnal, there appears to be a substantial degree of plasticity to this behavior (McDonough & Loughry, 1997). Armadillos can switch between nocturnal and diurnal activity seasonally, geographically, ontogenetically, or in response to local weather conditions (Harmsen et al., 2011; Loughry & McDonough, 2013; McDonough & Loughry, 1997). However, the aforementioned explanations did not appear to drive the patterns we were observing. Rather, it appeared as if armadillos at the most remote sites were more diurnal, while armadillos at sites closer to the urban center were more nocturnal. Our goal here was to evaluate if armadillo diel activity patterns were influenced by human activity or development at study sites situated along an urban to rural gradient in Arkansas, USA.

## 2 MATERIALS AND METHODS

### 2.1 Study sites

Our study took place at 10 public or private properties in the vicinity of Fayetteville, Arkansas, USA (Table 1). We chose 10 study sites to represent a continuum of development across the region from urban‐dominated sites within the city of Fayetteville to those surrounded by contiguous forests and occurring >50 km from Fayetteville. All sites occurred in the Ozark Mountains ecoregion and occurred at similar elevations (363–534 m). All sites were primarily upland oak–hickory mixed hardwood forests, with the exception of Wilson Springs Nature Preserve which was lowland forest dominated by Green Ash (*Fraxinus pennsylvanica*). Eight of the 10 sites were public property and were open for human recreation. Two other sites were forest patches within neighborhoods. The first, Elkins, consisted of two adjacent and wooded private properties located in a low‐density rural neighborhood and the other, Hyland Park, was also a series of wooded and adjoining private properties in a medium‐density suburban neighborhood on the eastern edge of Fayetteville. While neither of the private locations used in this study receive recreational visitation by the public, both experience regular and heavy use by residents who conduct lawn maintenance, daily commuting back and forth, and other regular activities associated with home ownership. The study took place during the winter period of October 2020 through March 2021 to limit effects of seasonal changes in armadillo behavior.

**TABLE 1:** Number of camera trap nights, cameras deployed, nine‐banded armadillo (*Dasypus novemcinctus*) detections, and number of detections occurring during the day captured by wildlife game cameras at 10 study sites in the vicinity of Fayetteville, Arkansas USA

Site (size in ha)	Camera Nights (# cameras)	No. of armadillo detections (diurnal detections)	Mean time of nocturnal armadillo activity (min after sunset)	Avg. distance to Fayetteville (km)	Avg. estimated sound level (dB)
Devil's Den State Park (890)	391 (10)	68 (21)	177	40	3.16
Devil's Eyebrow Nature Preserve (1220)	355 (12)	25 (15)	143	61.5	2.30
Elkins (8)	699 (10)	31 (0)	173	19.8	3.23
Hyland Park (28)	713 (7)	347 (0)	394	4	11.13
Kessler Mountain Regional Park (250)	517 (15)	73 (3)	226	8	8.11
Lake Wilson (140)	198 (10)	18 (0)	230	9.9	3.00
Markham Hill (58)	674 (10)	251 (11)	378	3.8	11.60
Bear Hollow Nature Preserve (160)	1017 (15)	372 (218)	155	57.1	2.65
Sequoyah Woods (39)	372 (12)	14 (0)	358	3.4	11.91
Wilson Springs Nature Preserve (49)	257 (6)	10 (2)	195	6.3	14.34
Total	5193	1209 (270)	312	24	7.52

Note

Straight line distance to downtown Fayetteville and estimated anthropogenic sound level was calculated at each camera location and averaged for all cameras at each site.

### 2.2 Armadillo activity

We used motion‐triggered wildlife game cameras to document the presence and activity patterns of nine‐banded armadillos at each study site. We used a combination of Spypoint Force Dark (Spypoint Inc, Victoriaville) and Browning Strike Force (Browning, Morgan) model cameras. To determine camera locations, we overlaid each study site with a series of 150 m × 150 m grids. Each grid was assigned a number and we randomly chose grids in which cameras would be placed. No grid received more than one camera at a time although at some sites multiple cameras were placed within the same grid at different times of the year. We used this approach to ensure cameras were dispersed across the study sites. Within selected grids, we placed cameras in locations with clear lines of sight for at least 15 m and in locations that would not be visible from roads or trails to avoid theft or the photography of people. We placed cameras on either trees or tripods 50 cm above the ground. We programmed cameras to operate 24 h per day, take bursts of three photos at each trigger, and to reset after 5 seconds. We downloaded memory cards from cameras approximately every 2 weeks. Due to constraints on the number of cameras, cameras were not deployed at all sites simultaneously and not all sites had equal sampling effort. At each specific location where a camera was deployed, we recorded the coordinates so we could later use a GIS (ArcMap 10.2; ESRI Inc) to calculate landscape variables around each camera.

We reviewed all photographs using the Timelapse 2.0 software (Greenberg et al., 2019). Timelapse 2.0 allowed us to rapidly review photographs, extract metadata (date, time), and to assign species identity to each group of photographs. Timelapse 2.0 also allowed us to group photographs into sequences. We assumed all photographs taken within 5 min of one another were one sequence of the same individual and these photographs were combined into one unique detection to reduce double‐counting individuals. We chose 5 min for the sequence length because most detections of armadillos foraging in front of cameras lasted for less than 5 min and allowed even slow‐moving individuals to move out of range of the camera. We scored each armadillo detection as occurring during day or night based on the sunrise and sunset time for the 15th day of that month so that our definitions of diurnal and nocturnal activity varied across the study to account for changing daylengths. We used the time of the first armadillo detection within the sequence to assign the timing of activity.

### 2.3 Analyses

To evaluate how armadillo daily activity patterns were influenced by human development and activity, we calculated several variables around each camera location using a GIS. We used currently available Landsat 8 imagery (Landsat 8; U.S. Geological Survey: https://earthexplorer.usgs.gov/ accessed 4/13/2021) to calculate the amount of forest cover (ha) within 500 m of each camera location. Similarly, we calculated the amount of development (ha) around each camera by summing the area containing either developed landcover or pavement occurring within 500 m of each camera.

Because we did not have access to daily visitation data for each study site, we developed two variables to serve as proxies for human activity: Euclidean distance to the nearest population center (i.e., downtown Fayetteville, Arkansas, USA) and estimated anthropogenic sound levels. Euclidean distance to nearest population center is an established index of urbanization related to human activity (Kellner et al., 2017; Moll et al., 2019). We measured the distance from downtown Fayetteville (36.066166, −94.157889) to each individual camera location. We assumed human visitation would increase with decreasing distance to Fayetteville. We used a US national 250‐m^2^ resolution geospatial data layer of estimated anthropogenic sound levels as predicted by Mennitt and Fristrup (2016). To create these data, acoustic recordings from 2000 to 2014 scattered across the United States in urban and rural areas were used as response variables for a random forest machine learning algorithm, and 45 landscape and environmental variables were used as predictors (Buxton et al., 2017). Anthropogenic sound levels were calculated by systematically minimizing contributions from all anthropogenic model inputs, leaving only biotic and abiotic sources of sound. Here, we used the “L50” anthropogenic sound level estimate, which is defined as the sound level exceeded 50% of times during an average summer daytime hour. We assume that higher levels of L50 sound correspond to higher human presence and activity in an area. Although this sound layer estimated sound levels during the summer, it has been used for studies exploring animal response to noise in the winter (Wilson et al., 2021) and Buxton et al. (2017) noted that this layer is valid for winter use but that overall sound levels may be lower in winter. We extracted the L50 sound value for the location of each camera.

To relate the timing of armadillo activity to the measured variables, we used generalized linear mixed models with armadillo activity period as a binomial response variable. We created the activity period by categorizing each armadillo detection as occurring during the day (after sunrise and before sunset) or during the night (after sunset and before sunrise). We explored the effects of the fixed factors: forest cover, urban development, month, distance to downtown, and sound level on the probability that armadillo activity would occur during the day versus night. To account for variation across sites, we used study site as a random effect. We assessed all variables for normality and appropriate levels of variance by creating plots of fitted and observed values and residuals and qqmath plots (Pinheiro & Bates, 2000; Sarkar, 2008). All analyses were conducted in SAS 9.4 (SAS Institute).

To better explore the magnitude of armadillo activity changes, we used regression analyses. We focused only on the timing of nocturnal activity to test the prediction that armadillos at busy human sites would be active later at night than those occurring at less human‐visited sites. For analyses, we removed all armadillo detections occurring before sunset or after the sunrise period. We then calculated the difference in time (min) of each armadillo detection and the reported sunset time for that date. Thus, an armadillo first detected 1 min after sunset was given a value of “1” and an armadillo detected 2 h after sunset was given a value of “120”. We then used a general linear model to explore the relationship between the time of nocturnal armadillo detections with each factor identified as important in the previous analysis from among forest cover, distance to downtown Fayetteville, and estimated sound level.

## 3 RESULTS

From October 2020 to March 2021, we recorded 1209 armadillo detection sequences (hereafter “detections”). Armadillos were detected at all 10 study sites. We had a total of 5193 trap nights that ranged from 198 to 1017 at each site (Table 1). The number of armadillo detections per site ranged from 10 (Wilson Springs) to 372 (Bear Hollow).

**FIGURE 2:**
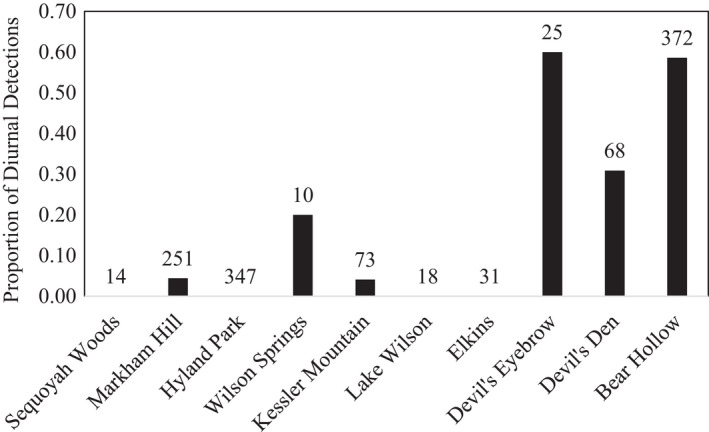
Proportion of nine‐banded armadillo (*Dasypus novemcinctus*) detections occurring during the day at each of 10 study sites in Arkansas, USA between October 2020 and March 2021. The number above each bar indicates the total number of armadillo detections at that site. Sites are arrayed in order of closest to downtown Fayetteville (Sequoyah Woods) to farthest (Bear Hollow)

Armadillo activity occurred both during the day and night. Overall, 22% (270 of 1209) of armadillo detections occurred during the day. However, the extent of diurnal armadillo activity varied considerably between sites (Figure 2). We recorded exclusively nocturnal activity at four of the 10 study sites, and at another 2 study sites more than 95% of armadillo detections occurred during the night. Conversely, at the three sites located farthest from Fayetteville—Bear Hollow, Devil's Eyebrow, and Devil's Den—diurnal armadillo activity was relatively common (60%, 59%, and 31%, respectively).

**FIGURE 3:**
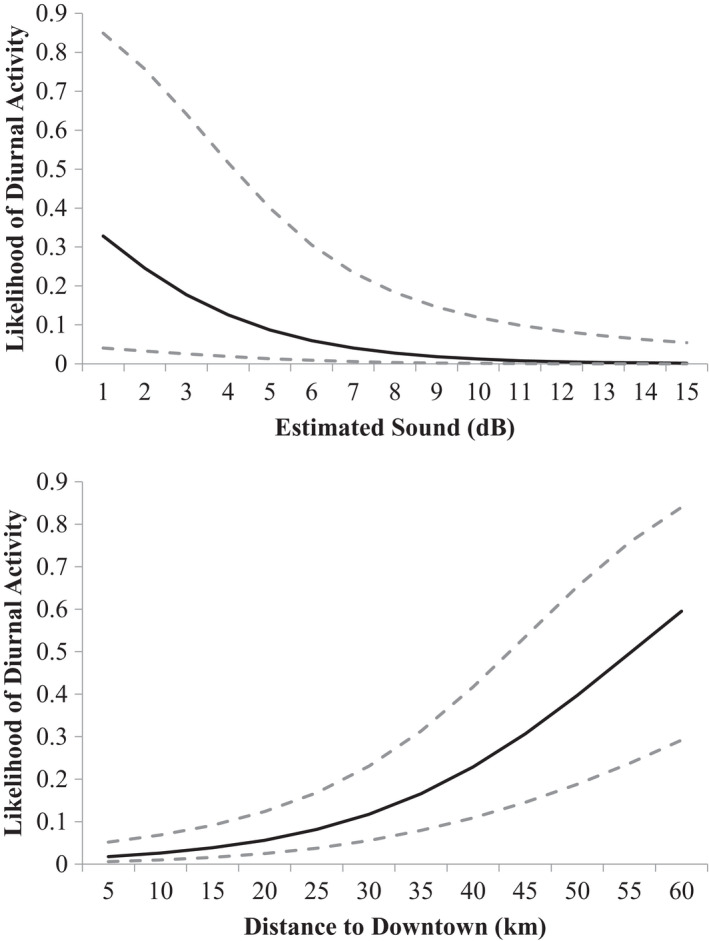
Probability (± 95% confidence intervals) that a detection of a nine‐banded armadillo (*Dasypus novemcinctus*) would occur during the day based on the effect of estimated sound (top panel) in the vicinity of each camera and the distance from each camera to downtown Fayetteville, Arkansas USA (bottom panel) between October 2020 and March 2021

**FIGURE 4:**
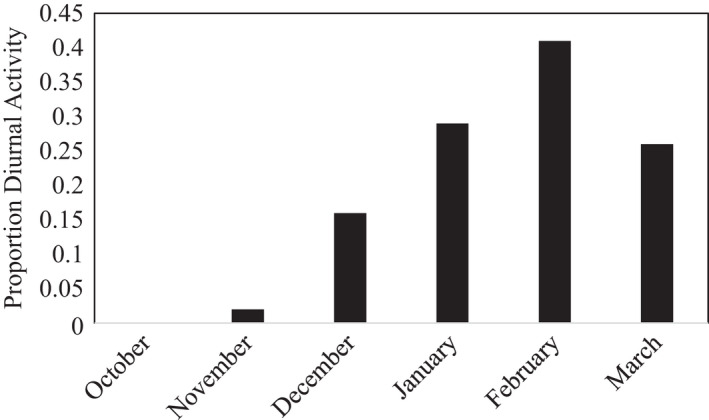
Proportion of nine‐banded armadillo (*Dasypus novemcinctus*) detections occurring during the day by month of the winter period between October 2020 and March 2021 in Arkansas, USA

The probability that an armadillo detection would occur during the day was most influenced by the estimated sound level around a particular camera location (*F*_1, 1198_ = 6.2, *β* = −0.408, *p* = .013; Figure 3). Diurnal activity was also negatively associated with distance to downtown such that sites far from Fayetteville were more likely to have diurnal armadillo activity, whereas those occurring close to Fayetteville were unlikely to have diurnal activity (*F*_1, 1198_ = 24.7, *β* = 0.08, *p* =.001; Figure 3). There was also a seasonal component to diurnal behavior (*F*_5, 1194_ = 8.09, *β* = −3.69, *p* = .001) with an increase in diurnal behavior as the winter progressed and presumably became colder (Figure 4). There was no relationship between diurnal armadillo activity and urban development (*F*_1, 1198_ = 1.11, *β* = 0.14, *p* = .29) and only a marginal relationship between diurnal activity and forest cover (*F*_1, 1198_ = 3.04, *β* = −0.032, *p* = .09).

**FIGURE 5:**
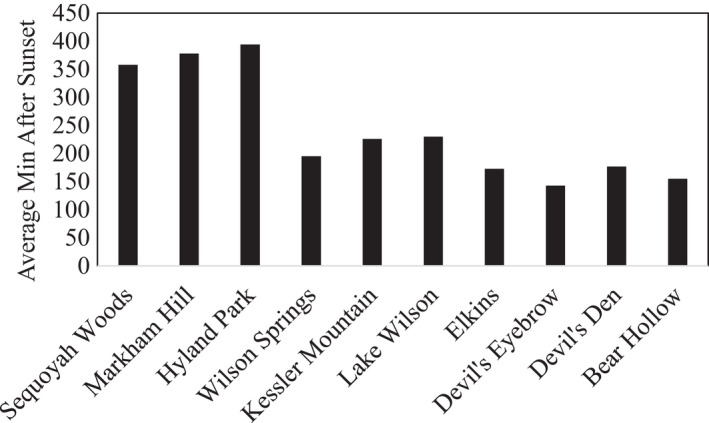
Average time after sunset that nine‐banded armadillo (*Dasypus novemcinctus*) activity occurred at each study site arranged in order of in order of closest to downtown Fayetteville (Sequoyah Woods) to farthest (Bear Hollow)

Our analyses of the timing of nocturnal armadillo activity indicated that the time of night that armadillos were active (minutes after sunset) varied significantly (*F* = 117.21, *df* = 4, *p* < .001). Armadillo activity was likely to occur later at sites with higher estimated sound levels (*F* = 44.64, *p* < .001), at sites closer to downtown Fayetteville (*F* = 349.28, *p* < .001), and in areas with less forest cover (*F* = 52.25, *p* < .001). Average time of nocturnal armadillo activity varied from 143 min (± 84) at Devil's Eyebrow to 394 min after sunset (± 246) at Hyland Park (Figure 5).

## 4 DISCUSSION

Our results show that armadillos likely shift to nocturnal activity in the presence of human activity and remain relatively diurnal in areas with little human activity (Figure 2). This behavioral plasticity displayed by the armadillo aligns with many widespread mammal species that occur in both natural and human‐dominated landscapes (Gaynor et al., 2018). While armadillos are noted for flexible diurnal activity patterns (McDonough & Loughry, 1997), this is the first study to link this behavior with the presence of human activity. However, there was one study that showed that armadillos in large Amazonian rainforest patches (>1000 ha) had different activity patterns than those inhabiting smaller patches, indicating that forest fragmentation can influence when armadillos are active (Norris et al., 2010). Pardo et al. (2021) showed that nine‐banded armadillos at their study sites in palm plantations were strictly nocturnal but there was no comparison with reference sites to determine if this was in response to human activity. While we lacked a direct measure of human activity at our different sites, both indices for human activity (distance to downtown and estimated sound) were strongly associated with the timing of armadillo activity (Figures 3 and 5). However, armadillos at Wilson Springs, a site located near downtown Fayetteville and with high estimated sound levels, showed more diurnal behavior than predicted. This may be because dogs are not allowed at this site and we believe dogs may play a role in diurnal armadillo behavior (see below). Alternatively, this may simply be an artifact of limited sampling at this site as we only detected 10 armadillos during 257 trap nights.

The shift to a primarily nocturnal existence in the presence of humans almost certainly has a fitness cost for armadillos. Armadillos have low body temperatures, low basal metabolic rates, and high thermal conductance (McNab, 1980). Physiologically, armadillos respond to cold temperatures by reducing their activity and their body temperature. Armadillos exposed to cold may take 3–4 days to re‐establish normal body temperature (McNab, 1980). Our study took place during the winter period in a montane climate that should be thermally challenging for armadillos, particularly during nocturnal periods. Armadillos foraging at night are exposed to suboptimal temperatures and likely have reduced access to their invertebrate prey which burrow deeper into the substrate in response to colder temperatures (Sikes et al., 1990). How this behavior affects their physiology, ability to acquire needed resources (e.g., foraging time), overlap with the availability of their invertebrate prey, or whether it alters their vulnerability to other predators is unknown and requires further exploration.

Although we showed armadillos altering behavior in response to human activity, our results do not elucidate causal mechanisms for this behavioral change. One potential mechanism is fear: multiple taxa have been shown to select habitat and modify behavior in response to “landscapes of fear” of humans and other predators (Kohl et al., 2018; Laundré et al., 2010). While the urban–rural behavioral shift we observed could be a fear response to the sound or sight of people, it could be related to disturbances that accompany human activity, namely, domestic dogs (*Canis lupus familiaris*). Dogs have been shown to have a profound influence on wildlife behavior and activity patterns (Gálvez et al., 2021; Zapata‐Ríos & Branch, 2016). For example, bobcats (*Felis concolor*) became more nocturnal in response to human activity and the presence of dogs (George & Crooks, 2006). In fact, the southern long‐nosed armadillo (*Dasypus hybridus*) has been shown to avoid farms with large numbers of dogs (Abba et al., 2007). During the course of this study, we witnessed firsthand several off‐leash dog attacks on armadillos and interacted with several dog walkers who relayed to us that their dogs had just attacked armadillos on their walk. Armadillos were diurnal at Bear Hollow Nature Preserve (59% of detections during the day) and this was one of two public study sites where dog walking was prohibited. The other site that prohibits dog walking is Wilson Springs, where despite limited sampling, armadillos were more diurnally active than anticipated based on distance to downtown and estimated sound. We documented numerous off‐leash dogs at both of the private sites in this study and adjacent landowners at both sites also owned dogs. Dogs are often considered hyper predators, and even if they do not fatally harm wildlife, the chasing response can be enough to trigger strong antipredatory behaviors in affected individuals (Zapata‐Ríos & Branch, 2016).

This report adds to the growing body of evidence showing nonlethal effects of humans on wildlife. These subtle effects are likely pervasive on much of the wildlife community and provide valuable information to managers of nature reserves near population centers. Enforcement of leash laws or prohibiting dogs from trails has been shown to be effective for changing the distribution of wildlife (e.g., Parsons et al., 2016). Wildlife have also been shown to benefit from the closing of parks to human visitation on certain days of the week (e.g., Whittington et al., 2019). Both land managers and researchers could benefit from further investigation and quantification of the costs of nonlethal disturbance to wildlife and by exploring mitigation measures to alleviate these costs.

## CONFLICT OF INTEREST

The authors have no conflict of interest to report.

## AUTHOR CONTRIBUTION

**Brett A. DeGregorio:** Conceptualization (lead); Funding acquisition (lead); Investigation (lead); Project administration (lead); Resources (lead); Writing‐original draft (lead). **Caleb P. Roberts:** Formal analysis (supporting); Methodology (supporting); Writing‐review & editing (supporting). **Ellery Lassiter:** Formal analysis (supporting); Writing‐review & editing (equal). **John Veon:** Investigation (supporting); Writing‐review & editing (supporting). **Andrhea Massey:** Data curation (supporting); Formal analysis (supporting); Visualization (supporting); Writing‐review & editing (supporting). **Connor Gale:** Writing‐original draft (supporting); Writing‐review & editing (supporting).

## Data Availability

Data are available on Dryad https://doi.org/10.5061/dryad.d7wm37q2d
